# A computational model of organism development and carcinogenesis resulting from cells’ bioelectric properties and communication

**DOI:** 10.1038/s41598-022-13281-3

**Published:** 2022-06-02

**Authors:** Joao Carvalho

**Affiliations:** CFisUC, Department of Physics, University of Coimbra, Coimbra, Portugal

**Keywords:** Computational biophysics, Cancer models, Numerical simulations, Computational models

## Abstract

A sound theory of biological organization is clearly missing for a better interpretation of observational results and faster progress in understanding life complexity. The availability of such a theory represents a fundamental progress in explaining both normal and pathological organism development. The present work introduces a computational implementation of some principles of a theory of organism development, namely that the default state of cells is proliferation and motility, and includes the principle of variation and organization by closure of constraints. In the present model, the bioelectric context of cells and tissue is the field responsible for organization, as it regulates cell proliferation and the level of communication driving the system’s evolution. Starting from a depolarized (proliferative) cell, the organism grows to a certain size, limited by the increasingly polarized state after successive proliferation events. The system reaches homeostasis, with a depolarized core (proliferative cells) surrounded by a rim of polarized cells (non-proliferative in this condition). This state is resilient to cell death (random or due to injure) and to limited depolarization (potentially carcinogenic) events. Carcinogenesis is introduced through a localized event (a spot of depolarized cells) or by random depolarization of cells in the tissue, which returns cells to their initial proliferative state. The normalization of the bioelectric condition can reverse this out-of-equilibrium state to a new homeostatic one. This simplified model of embryogenesis, tissue organization and carcinogenesis, based on non-excitable cells’ bioelectric properties, can be made more realistic with the introduction of other components, like biochemical fields and mechanical interactions, which are fundamental for a more faithful representation of reality. However, even a simple model can give insight for new approaches in complex systems and suggest new experimental tests, focused in its predictions and interpreted under a new paradigm.

## Introduction

Life sciences need a theory of organisms to test different hypothesis and consequences of its fundamental premises. This theory would deal with the way an organism spontaneously grows from a single cell to a complete and complex organism, introducing organization at the different development stages and homeostasis for a mature organism. The introduction of such description is a hugely ambitious aim, akin to a Theory of Everything^1^, in Physics, or the General Theory of Employment, Interest and Money^2^, in Economy. An organism’s growth and transformation during its life cycle is a tremendously complex succession of processes and events but even a simplistic and limited theory, dealing with only the general trend and generating a broad vision of tissue organization, can drive specific studies and experiments to further inform this field of research.

Theories of organism development have a long history and many contributions were proposed, starting from diverse premises and centering in different views. In his pioneering work, Jacques Loeb^3^ considered that physicochemical biology completely and correctly explained the development of organisms and all participating processes. James Miller introduced the living systems theory^4^, where each system must include a number of critical subsystems, dealing with their structure, interaction, behavior and development, that can be applied from simple cells to full organisms. Robert Rosen^5^ rejects the reductionist perspective of an organism as a mechanism, which ignores the full extent of organism complexity. He defended that complex organization is a universal feature of all systems. On the same line of thought, another important contribution was expressed by Denis Noble noble2006, showing that organisms are much more than simple machines built from genetic information, and the relationship between parts is fundamental as they affect gene expression. A critical analysis of evolution, in particular some failings of simple Darwinism, was produced by Brian Goodwin^7^. A particular addition to the theory of organism development was his introduction of morphogenetic fields, adding spatial and temporal dimensions to the development of forms. A complementary viewpoint involves the contribution of bioelectricity to organism development, in particular in morphogenesis and organization^8–10^, which is also the modeling context for the present work.

A proposal of a Theory of Organisms (ToO)^11^ is being developed and presented in the last years by a committed group of researchers. Their proposition is based on three main principles: (a) Cells’ default state is proliferation and migration with variation (generation of novelty); (b) the principle of variation; (c) the principle of organization by closure of constraints (for generation of stability and robustness). The present work, a cellular automaton, introduces a simplified computational model based on some of the main premises of this ToO, to describe the growth of an organism from a single proliferative cell, and its organization, based only in the progression of the bioelectric state of cells and tissues, and spatial constraints. A cell is represented by a pixel in a two-dimensional domain, with certain properties, and its state evolves according to a set of rules. These are a function of the states of the cell and of its neighbors. Using very simple and straightforward rules, it is possible to describe the growth and homeostatic state of a hypothetical simple two-dimensional organism or tissue. This is attained by the evolution of the cells’ bioelectric state and their close communication with neighboring cells and extracellular medium. Other models concentrate in alternative effects, like physical forces in organogenesis reported in^12^ (also including the use of cell’s default state and the principle of organization by closure of constraints), or the mechanical behavior and gene regulation^13^, transcriptomics^14^ or bio-mechanical properties and interactions^15^. A cellular automaton modeling of a tissue, applied to the nervous system, was previously developed^16^, including invasion by neural crest cells, proliferation of adult neural stem/progenitor cells and lateral inhibition of embryonic stem cells. A review^17^ reports on the different techniques used in computational modeling of tissue growth and regeneration, focusing on discrete models. A more recent review^18^ describes and compares cell-based modelling approaches, from lattice-based cellular automata to lattice-free models, applied to self-organization of multicellular tissues.

The non-neural cells’ bioelectric characteristics are important for their properties and behavior. The membrane electric potential and ion fluxes in and out of the cells can regulate their features and patterns at the organ-level^19,20^. The evolution of the cell bioelectric state is involved in the patterning of muscle and heart^21^, left-right arrangement of an organism^22^ and in regeneration of tissues^23^. Several experimental results show that proliferative cells are, in general, depolarized (with the transmembrane electric potential, $V_m$, less negative than non-proliferative ones)^24–26^. Cells have several electric potential regulatory mechanisms, in particular ion channels and pumps^27^ in interaction with the extracellular environment, and gap junctions to communicate with neighbor cells^28–30^.

The simple model proposed in this work allows also to address the carcinogenesis problem, by using premises from the Tissue Organization Field Theory (TOFT)^31^. The excessive, poorly controlled and non-homeostatic cell proliferation can be due to a deregulation of the tissue bioelectric state, a consequence of depolarization events. This non-mutagenic incident^32–34^ implies that carcinogenesis can be due to a failure on tissue organization process and not the result of an individual cell failure, with cell mutations being a consequence rather than the cancer cause^35^. The cells’ bioelectric state was shown, by several experimental tests, to have influence in carcinogenesis and in cancer therapy^36^, with the most relevant factors being the transmembrane electric potential and the conductance through ion channels. A study about the connection between cancer hallmarks and ion channels pathologies was published^37^, showing that gap junctions’ performance is relevant in tumor development, in particular its long-range control^38^.

Michael Levin^39^ described how ion flow acts as an important epigenetic regulator of cell behavior. In his work it is pointed out by which means the bioelectric signaling is involved, at diverse spatial scales, in feedback loops, long-range communication and information transfer. Changes in cell membrane potential translates to modulation of canonical pathways involved in, among other morphogenic processes, cell proliferation, apoptosis, migration, orientation and differentiation. Alexis Pietak and Michael Levin^40^ describe how bioelectricity can be integrated in genetic and reaction networks, where $V_m$ can change the concentrations of key signaling molecules, both inside and outside the cells. In this work it is shown that $V_m$ controls biochemical signals involved in some fundamental feedback cycles. In a review by Sheena Tyler^41^, it is explained the role of current in morphology, as wound healing and regeneration driver. In a recent review^42^, Matthew Harris discusses how electric potentials are correlated with cell properties and tissue organization, and also provides evidence linking bioelectric signaling to development regulation. Many experimental observations point out to the importance of bioelectricity in organism development and regeneration, supporting the conclusion that the tissue’s bioelectric state is fundamental for a correct organism development. In numerous cases, bioelectric signaling, in particular cell membrane potential, controls biochemical pathways involved in morphogenesis. These results are the foundation of the present work and, even if bioelectrical properties and states are not alone in determining the organism outcome, they are considered dominant in some of the most relevant events in development”.

The next section explains, succinctly, the Theory of Organisms, and the biology behind the cell bioelectric state as well as its importance in a system growth. This is ensued by a depiction of the computational bioelectric model that was developed, and also the way carcinogenesis was introduced in the simulation. The results obtained in a number of tests are described, including the induction of carcinogenic events followed by simulation of cancer therapy. Finally, the obtained results are discussed in the context of the proposed theoretical framework, conclusions are produced, and possible experimental tests suggested.

## Theory of organisms

The ToO intends to explain, in a very general way, how organisms develop, achieve their intended size, shape and function, and keep their homeostatic behavior^11^. It also contributes to explaining some pathological events, as malformations and carcinogenesis. The hypotheses that will be used in the current work are being developed for some time by an international and multidisciplinary team of scientists, and are based on three general, evidence based, principles. These are that cells’ default state is proliferation and migration with variation^43^, the principle of variation^44^, and the principle of organization^11^.

The present model, when applied to tumor initiation, is related to the Tissue Organization Field Theory (TOFT)^31,45^, as it deals with the emergence of tissue organization at a supra cellular level, and cancer is considered a disease of tissue homeostasis regulation, not initiated from an individual cell pathological behavior but a tissue organization breakdown. Accordingly, a tumor originates from a perturbation on tissue environment and reciprocal interactions between parenchyma and stroma, not from a single mutated cell, leading to a transition from an organized to disorganized state. In this work, intercellular communication by bioelectric signals is a mechanism driving homeostasis at tissue level; however, after an extensive perturbation, the tissue bioelectric state can change and the cells’ properties be modified. This process can also be present during organism development (embryogenesis), where variations in spatial and temporal distribution of membrane electrical potential and ion fluxes can be responsible for the establishment of tissue abnormal patterns. It can also be involved in many fundamental processes, as regeneration of limbs, definition of the anatomical axes and/or formation of eyes^46^. With mature tissue cells being mainly in a polarized state (and then non-proliferative), when a depolarization (carcinogenic) event occurs it can, in certain circumstances, overwhelm the feedback systems responsible for control of tissue organization. In these conditions, cells can have their epigenetic program modified, due to modifications in their bioelectric state, leading to changes in genomic expression of relevant biochemical pathways. A strong bioelectric coupling with neighboring cells via gap junctions can drive, or block, the propagation of the depolarization state. The excessive cell proliferation creates adverse conditions for cells’ survival, which includes a decrement in nutrient concentration, hypoxia and reduced pH, promoting DNA damage, stimulating genetic mutations and reducing DNA’s repair capability^47,48^.

### A change of cell and tissue bioelectric state can alter biochemical pathways

The cell bioelectric state, in particular the transmembrane potential ($V_m$), is determined by the expression and activity of ion channels, ion pumps and gap junctions. The differential concentration of ions across membrane, like K$^+$, Na$^+$, Cl$^-$ or Ca$^{2+}$, leads to a usually more negative electric potential at the membrane intracellular side. These changes take place during the different cell cycle phases^49^. When $V_m$ is smaller than about − 40 mV the cell is considered polarized (or hyperpolarized), otherwise, with a less negative potential, is depolarized.

Throughout an organism’s development, the bioelectric state and properties of non-nervous cells change appreciably, with precise spatial and temporal modifications on transmembrane electric potential values^8^. A cell can be in a polarized (or hyperpolarized) state, when $V_m$ is on the interval between $\simeq -50$ and $\simeq -90$ mV, or in a depolarized state, with $V_m\simeq -10$ to $\simeq -30$ mV. In the first case, the cell presents a quiescent behavior; in the other condition, cells are, predominantly, in a proliferative state^50^. This result was an important motivation for the present work, with depolarized cells being involved in the first stages of organism development, driven by cells’ high proliferative capacity, and also at the origin of transition from a normal to a tumor tissue^49^. Both proliferative cells in an adult organism and cancer cells are, in general, depolarized. This state can drive the particular behavior of a tumor cell, including uncontrolled expansion, greater motility and invasion capacity^51^. A change of cell polarization state can influence the expression of some genes, like Frizzled, which is controlled by a $V_m$ gradient^36^. A change of membrane voltage, in particular its depolarization, translates into transcriptional modifications, as the nuclear location of Yap/Taz, which correlates with spatial growth patterns^52^. It was also shown that cell’s depolarization induces the Rac1 activation, resulting in a motile phenotype^51^. Then, in these conditions, there is no change on genome sequence to modify a particular gene expression, but just a change in the transmembrane voltage^53^. In the present model it is not considered the bioelectric interaction between the epithelium tissue and stroma. This communication was shown to be involved in carcinogenesis^54^, that can be due to abnormal epithelial-stromal interactions or anomalous transcriptional activity of cytoplasmic Connexin43^55^.

Genetic instability^47^ can be a result of inadequate or overwhelmed tissue organization mechanisms, generated by environmental stressful conditions, like hypoxia, created by excessive and unregulated cell proliferation. The increased mutation rate found in some tumor tissues can, therefore, be a consequence of tumor progression, and it is not necessarily at its origin^34^.

The computational model of organism development presented in the following section is rooted in this biological input. The correlation found between cell membrane bioelectric depolarization and its proliferation capacity is used in tissue and organism growth, both in a healthy process and in carcinogenesis. This last outcome is explained not from a succession of mutations but from a disruption of bioelectric regulation, leading to abnormal cell depolarization. Related with this is the knowledge, still incomplete, that not only genetic information determines the cell membrane potential but also the opposite is valid, that the value of $V_m$ controls some relevant genetic pathways.

## Methods: computational model

The expansion and stability of a generic two-dimensional tissue, a single layer of cells, was implemented in a computational model, a cellular automaton, to describe the dynamics and organization of a hypothetical organism. The cells’ bioelectric properties and state are responsible for coordination of development and homeostasis, and they follow approximately the model developed by Cervera et al., described in detail in^56–58^, and adapted and used in a previous work^59^. Cells can exchange ions with the extracellular medium by collective depolarization and polarization ion channels, and the intercellular communication is described by a universal gap junction. The activity of each of these components depends on the membrane voltage, as shown in the following equations. The electrical potential (relative to the cell exterior) of cell *i*, $V_i$, changes due to ionic currents (considering only the migration of positive ions). Its variation with time is given by the differential equation,

1 $$\begin{aligned} C_i \frac{dV_i}{dt} = -I_{\text {pol}} -I_{\text {dep}} + \sum _j^{\text {neigh.}} G_{ij}\left( V_j - V_i\right) \end{aligned}$$

where $C_i$ is the cell membrane capacity, $I_{\text {pol}}$ ($I_{\text {dep}}$) the current through polarization (depolarization) ion channels and $G_{ij}$ the conductance of ion transfer through gap junctions between contiguous cells *i* and *j*. The sum is run over the 4 closest neighbor cells, if present (von Neumann neighborhood). The currents and the conductances depend on the membrane electric potential as follows,

2 $$\begin{aligned} I_{\text{ pol }}= & {} \frac{G^0_{\text{ pol }} \left( V_i - E_{\text {pol}}\right) }{1+\exp [z\left( V_i + V_T\right) /V_T]} \qquad , \qquad I_{\text{ dep }} = \frac{G^0_{\text{ dep }} \left( V_i - E_{\text {dep}}\right) }{1+\exp [-z\left( V_i + V_T\right) /V_T]} \end{aligned}$$

3 $$\begin{aligned} G_{ij}= & {} \frac{2G_{ij}^0}{1+\cosh [\left( V_i - V_j\right) /V_0]} \end{aligned}$$

with

4 $$\begin{aligned} G_{ij}^0 = \frac{G_{i}^0 G_{j}^0}{G_{i}^0 + G_{j}^0} \qquad \text {and}, \qquad G_{i}^0 = \frac{G_{\text{ max }}^0}{1+\exp [z\left( V_i - V_{1/2}\right) /V_T]} \end{aligned}$$

where $E_{\text{ pol }}$ ($E_{\text{ dep }}$) is the polarization (depolarization) membrane electric potential, $V_T$ the threshold potential, *z* the channel gating charge, $G^0_{\text {pol}}$ ($G^0_{\text {dep}}$) the polarization (depolarization) ion channels conductance, $V_{1/2}$ the value of membrane electrical potential that reduces $G_{i}^0$ by a factor 2, and $V_0$ adjusts the gap junction conductance function width^58^. The model parameters were determined, based on experimental results, and presented in^58^ for a generic non-excitable cell; for the current work they suffer minor adjustments and are given in Table 1. From these parameter values, the cells’ electric potential tends to $\simeq -57$ mV in a polarized state and to $\simeq -2$ mV in a depolarized one, with the separation between these two stable points at $\simeq -35$ mV. As previously shown, $G_{ij}$ describes the gap junction ion conductance between adjacent cells, representing the results of the serial association of cells *i* and *j* connexons conductances ($G_{i}^0$ and $G_{j}^0$)^60^. Different conductance values are introduced for the polarization, depolarization and gap junction channels for polarized and depolarized cells (through the magnitude of the parameters $G^0_{\text{ pol }}$, $G^0_{\text{ dep }}$ and $G^0_{\text{ max }}$). In this way it is possible to describe the ion flux dynamics of cells with the exterior and with other cells, which can change the membrane potential and cell polarization state (and then its phenotype and respective behavior).

**Table 1:** Values of the cells’ bioelectric parameters used in the present model.

Parameters	Mean value	Standard deviation
$C_i$ (pF)	100	$0.02 \sigma C_i$
*z*	3	0.1
$G_{\text{ ref }}^0$ (nS)	1	0
$G^0_{\text{ pol }}$ (polar./depol.)	$2.0 G^0_{\text{ ref }}/0.5 G^0_{\text{ ref }}$	$0.02\sigma G_{\text{ pol }}^0$
$G^0_{\text{ dep }}$ (polar./depol.)	$1.7 G^0_{\text{ ref }}/2.0 G^0_{\text{ ref }}$	$0.02\sigma G_{\text{ dep }}^0$
$G^0_{\text{ max }}$ (polar./depol.)	$4.0 G^0_{\text{ ref }}/2.5 G^0_{\text{ ref }}$	$0.02\sigma G_{\text{ max }}^0$
$E_{\text{ dep }}$ (mV)	0	$\sigma$
$E_{\text{ pol }}$ (mV)	− 60	$\sigma$
$V_T$ (mV)	26	$\sigma$
$V_0$ (mV)	24	0
$V_{1/2}$ (mV)	0	$\sigma$

The standard values for polarized and depolarized cells are different for some of the parameters. $\sigma =1$ is used as the standard deviation of normally distributed values around the mean value (stochastic model).

Cells are not precisely indistinguishable and, in this model, the principle of variation is introduced with the main individual cell bioelectric parameters magnitude following a normal distribution. In Table 1 the second column presents the parameter mean value and the third column the respective standard deviation.

## Carcinogenesis

The process of cancer initiation, i.e. carcinogenesis, can be better explained by an alternative theory to the mainstream Somatic Mutation Theory (SMT)^61^. TOFT describes the transition from normal to abnormal behavior in tumorigenesis as being due to a collapse of tissue organization mechanisms, leading to poorly controlled cell proliferation (expressing cells’ default state)^31^. This not only outlines the regulation of organism development by the bioelectric state of cells and tissues (an organizing field), as described in experimental works^8,30,46,50,62,63^, but also explains carcinogenesis without resorting to genetic mutations^36,38,64,65^. The hypothesis is testable and simple, and, with additional experiments, can be improved and extended to other pathologies. An event, being due to either ionizing radiation, a carcinogenic chemical substance, or any other process, changes the tissue homeostasis and cells can express back their default state, which is proliferation and migration^66,67^. In the present computational model, these carcinogenic events will be introduced by one of two incidents, namely, either the induction of a depolarized circular spot, as the result of localized carcinogenesis, or the random depolarization of a certain percentage of the tissue cells, a diffuse event. In the first case, a circular patch of depolarized cells, with $E_{\text{ dep }}=0$ mV and the bioelectric parameters corresponding to depolarized cells, is introduced. In the second experiment, the depolarized cells are randomly distributed on the tissue. In both cases, the evolution in time of cell membrane electric potential follows Eq. (1).

Carcinogenic events, such as a hit by ionizing radiation, the diffusion of a chemical substance, the introduction of extraneous materials^68^, or any other relevant perturbation on tissue organization, can induce cell depolarization^69^. The radiogenic activation of Ca$^{2+}$-permeable cation channels and of Ca$^{2+}$-activated K$^+$ channels, are examples of these kind of events. This shows the capacity to change the cell bioelectric state and can contribute to cell death^70^. It was also reported that K$^+$ channels can be activated by ionizing radiation^71^. These events usually affect a significant number of cells, not just one of them, turning out to be a tissue wide event. Nevertheless healthy tissues are, in general, resilient to this type of events, as the bioelectric communication among adjacent cells, through gap junctions’ channels^28^, drive ion concentration normalization (a community effect) and returns cells bioelectric state to its normal polarization condition^58^.

In a normal situation, in the current proposal, the system is highly resilient and if the perturbation extension is not large enough, if the percentage of affected cells is small and/or if it is not concentrated in an extensive region, the bioelectric communication among adjacent cells returns them to their previous state of polarization (non-proliferative cells). Otherwise a depolarization region can develop, with the consequent cell proliferation capacity and tissue growth, where there is space available.

This novel model can explain hereditary cancers^72^ by the cells’ bioelectric variability, in particular the expression of genes related with ion channels and pumps, and of connexins, which translates into different individual sensibilities to carcinogenic events. This is implemented in the model by the initial cell bioelectric properties and how fast a depolarization event can occur later in the organism life. It is well known that cancer is much more probable in old age^73^, and this is explained by a diminished bioelectric resilience, due to previous depolarization events, which leaves some cells more sensitive, increasing the probability of a depolarization transition.

An inherent consequence of the presented model is that tissue depolarization can be reversed, and hence the tumor growth may be contained by therapies that target the cells’ bioelectric state. As shown in^9^, the therapy can be implemented by stimulation of the potassium/sodium hyperpolarization-activated cyclic nucleotide-gated ion channel 2 (HCN2), in pursuance of the hyperpolarization and/or depolarization of different tissue regions. This intervention in brain teratogenesis drives the return to normal gene expression^19^. It was shown^63^ that small molecule drugs can be used to alter the ion channels properties, leading to an adjustment of the membrane electric potential. In case of undesirable activity, it can be used to restore their activity.

## Model results

The square domain, in 2D, represents empty space, where a tissue or hypothetical organism can be formed, by cellular proliferation^62^, starting from a single depolarized/proliferative cell (a depolarized cell is considered proliferative as opposed to a polarized one). Cells can also, with some probability (1%), move to an empty neighbor space, and, likewise, can die (with probability $1\times 10^{-4}$). After each mitosis a cell changes its bioelectric properties, moving in the direction of a polarized/non-proliferative state. But it retains its proliferative potential, which can be revived by environmental changes (like a neighbor cell death) or by an external action (such as a carcinogenic event). Cells don’t have exactly the same bioelectric properties (bioelectric parameter values), but show variations around a central value (presented in Table 1), truthful to the principle of variation.

A standard cellular automaton run, in a tissue growth process, is initiated by a single depolarized (and then proliferative, expressing its default state) cell placed at the domain center. This initial cell, in successive simulation runs, has an average membrane electrical potential $V_m$ centered at $E_{\text{ dep }}=0$ mV, distributed according to a gaussian function with $\sigma =1$ mV. The domain contains $300\times 300$ spaces. As bioelectric mechanisms are much faster than the other relevant biological processes considered in this model (like proliferation, migration or death), the simulation is run in an iterative way until it converges (maximum change of any cell membrane potential between successive iterations below 0.1 mV). After this convergence, the proliferative cells (the depolarized ones, with $V_m>-10$ mV), like the initial one, undergo mitosis if they have an adjacent free space. If it multiplies, the two resulting cells have slightly changed bioelectric parameters, moving in direction of the values characteristic of polarized cells. After each mitosis the new cells’ bioelectric parameters vary around the corresponding progenitor cell values (gaussian distributed, with the same value of $\sigma$ as used before, faithful to the principle of variation in the system evolution). But the values of $G^0_{\text{ pol }}$ and $G^0_{\text{ max }}$ of the daughter cells increase by 2% with respect to the progenitor, and the value of $G^0_{\text{ dep }}$ decreases by 2% (these are the standard rates; other values were tried and will be shown in the “Model results” section); this happens in each cell proliferation until the parameter values reach the corresponding ones of standard polarized cells (values shown in Table 1)^74^.

### Depolarized cells concentrate at the tissue center in homeostatic conditions

**Figure 1:**
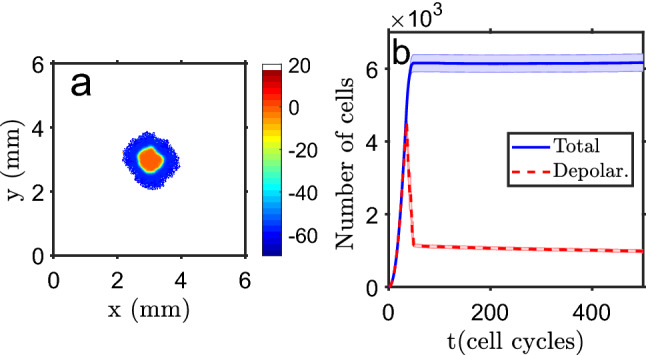
Standard simulation run. (**a**) Example of the final state of the system, with the color code showing the bioelectric state of each cell (membrane potential): a depolarized core surrounded by polarized cells, with a narrow transition border of cells with intermediate values of $V_m$ (scale in mV). Each cell is represented by a 20$\mu$m size square pixel. (**b**) Evolution in time (in units of cell proliferation cycles) of the total number of cells (blue line) and the number of depolarized cells (dashed red line). At $t\simeq 40$ cycles a fast transition to tissue polarization occurs (except for the depolarized core) and an approximately constant number of cells is reached (homeostasis). The bands show the standard deviation of the mean of $n=25$ simulation runs.

Depolarized cells, like tumor cells, have lower bioelectric connectivity^75–77^ with the neighbor units due to a reduced gap junction activity^78,79^. The tissue grows until it reaches a homeostatic state, as shown in Fig. 1a, with a depolarized core (proliferative cells), where cells cannot multiply for lack of space, surrounded by polarized ones (non-proliferative while in this bioelectric state). Cells at the tissue rim have exhausted their proliferation potential due to successive steps in the direction of polarization. The system reaches a quasi-stable state, where cells at the periphery can move a bit and cells in the core can proliferate or move only when a neighbor cell dies. Some random cell migration occurs at the edges (but cells that get fully detached from the main mass are discarded) and cells have a specified death probability. When a cell dies, one of its neighbor cells depolarizes and, in general, is able to proliferate and fill the gap, keeping the number of cells in the tissue approximately constant. The interface between depolarized core and polarized rim has a width of about 8 cells, which show intermediate values of $V_m$. Figure 1b presents the change in time of the total number of cells and of depolarized cells, showing a fast transition from mainly depolarized cells to a essentially polarized domain, with depolarized cells just in the core. The main simulation time step is the average time between two successive proliferation events (cell cycle). The size of the tissue is mainly determined, as will be shown later, by the cells’ polarization rate after each mitosis cycle.

### A wound is repaired by proliferation of cells at tissue core

**Figure 2:**
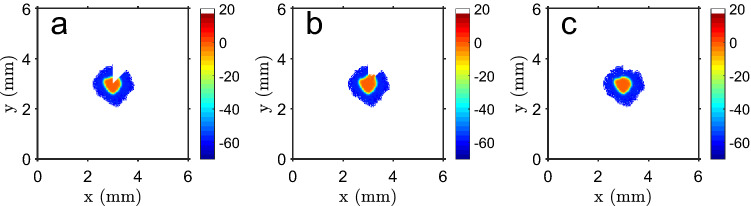
Wound healing test. (**a**) Example of a wound healing test, when about of 1/8 of the cells are removed after 300 cycles. (**b**) Ten cycles after the injury, cells from the depolarized core proliferate into the empty space, starting to fill it again. (**c**) After the test ($t=500$ cycles), the tissue recovered its shape and most of the lost cells. Color bar: cell membrane potential, in mV (white shows empty space).

In a test of system homeostasis, a wound healing test was performed by removing about 1/8 of the cells at $t=300$ cell cycles, and check the tissue recovery for 200 cycles. It is shown in Fig. 2 that the system repairs the injury in a fast way, closing the gap and restoring the initial shape and size.

### Computational model tests show effects of aging and drugs in cells polarization state

**Figure 3:**
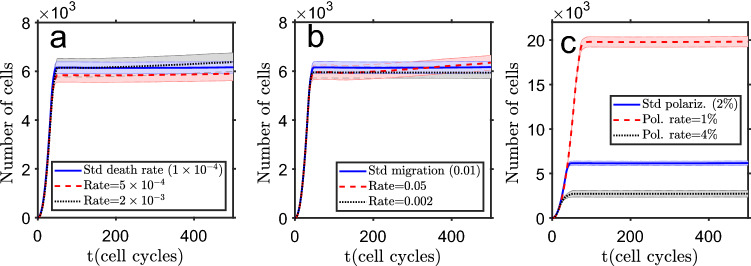
Test of model parameters. (**a**) Change of cells death rate. (**b**) Variation of cells’ migration probability. (**c**) Shift of cell polarization rate after proliferation. The bands show the standard deviation of the mean of $n=25$ simulation runs.

The computational model was run in many different conditions and the main results are now presented. Some of the parameters were changed by large (but reasonable) values, with the modified system evolution being, in general, what would be expected. In Fig. 3a, the standard cell death rate ($1\times 10^{-4}$ per cell cycle) was changed by a factor of five, up and down, with minor differences on the total number of cells (homeostatic state) observed, but with a decrease in the number of depolarized cells (concentrated at the core) with a higher cell death rate, due to exhaustion of their proliferation potential. This result can be related with the consequences of tissue aging. The effect of changing cell migration probability, from the standard $P=0.01$ to five times higher or lower (shown in Fig. 3b), also has almost no effect on the tissue size (cells fully detached from the main tissue mass are discarded). But changing the cells’ rate of polarization after proliferation (the rate at which their main parameters, $G^0_{\text{ pol }}$, $G^0_{\text{ dep }}$ and $G^0_{\text{ max }}$, get closer to the ones of a polarized cell) has, as expected, a substantial effect on the final size of the tissue, as shown in Fig. 3c. A slower polarization rate (1% instead of standard 2% per cell cycle) increases the maximum number of possible proliferation cycles, hence the homeostatic tissue size. The opposite occurs with a higher polarization rate (4%), with a decrease on the total number of cells and the disappearance of depolarized cells in the tissue.

### Variation of stochasticity level, cell communication and initial depolarization change the final tissue size

**Figure 4:**
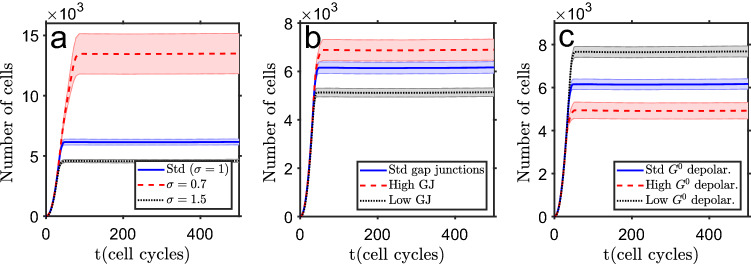
Test of model parameters. (**a**) Change of cell variability level $\sigma$ (standard deviation of the parameters’ central value). (**b**) Variation of the inter-cell communication level (gap junctions’ conductance). (**c**) Shift of initial cell bioelectric properties: high $G^0$ means closer to the polarized state. The bands show the standard deviation of the mean of $n=25$ simulation runs.

Another set of model tests involved an additional group of parameters. The first was the variation (stochasticity) level, the value of the standard deviation around each parameter central value. Increasing this variation (shown in Fig. 4a), drives the tissue to a smaller size. This result looks somehow counter intuitive but it follows from a larger dispersion of the cell parameters, which make cells more prone to initiate a polarization wave, starting at tissue periphery and stopping cells’ proliferation. Figure 4b shows the effect of modifying the level of inter-cell communication, by changing the gap junctions’ conductance. Decreasing this communication (from $G^0_{\text{ max }}=4G^0_{\text{ ref }}$ to $G^0_{\text{ max }}=3.6G^0_{\text{ ref }}$, and $G^0_{\text{ max }}=2.5G^0_{\text{ ref }}$ to $G^0_{\text{ max }}=2.3G^0_{\text{ ref }}$ for polarized and depolarized cells, respectively) reduces the system final size, as there is a weakened collective normalization effect by neighbor cells to retain the depolarization (in the higher communication test, these parameters where increased to $G^0_{\text{ max }}=4.4G^0_{\text{ ref }}$ and $G^0_{\text{ max }}=2.8G^0_{\text{ ref }}$ for polarized and depolarized cells, respectively). Finally, in Fig. 4c, it is shown how the number of cells evolves in time with a change of cells’ bioelectric parameters. As expected, when the depolarized cells have parameters closer to the polarized ones (high $G^0$ parameters, changing to $G^0_{\text{ pol }}=0.6G^0_{\text{ ref }}$, $G^0_{\text{ dep }}=1.8G^0_{\text{ ref }}$ and $G^0_{\text{ max }}=2.8G^0_{\text{ ref }}$, as compared with the ones given in Table 1), proliferation potential is reduced (reaches the polarized state after a lower number of proliferation cycles) and the system grows to a smaller size.

From the reported tests some conclusions can be extracted. The tissue reaches a homeostatic size and shape, with a depolarized core that can act as a proliferative cells’ reservoir (it will decrease naturally with time, as an effect of cumulative cell death)^80,81^. The proliferation of these cells can be used to replace dead cells or to repair a limited tissue injury. The equilibrium size of the tissue can be controlled by the rate of cell polarization after each mitosis and how close the initial depolarized cell bioelectric parameters are to the ones of a polarized cell. When they become polarized they enter into a non-proliferative state, unless there is an external depolarization event (or if a cell dies in the vicinity). The level of variability of the cell parameters is also very important for the final homeostatic state, as the polarization starts in a spot at the periphery and propagates in a fast way to all the domain outer rim. The faster a polarized spot develops (which is more probable with higher parameter variation), the earlier the proliferation stops and the smaller is the final system.

### Carcinogenesis can be induced by cell depolarization events

**Figure 5:**
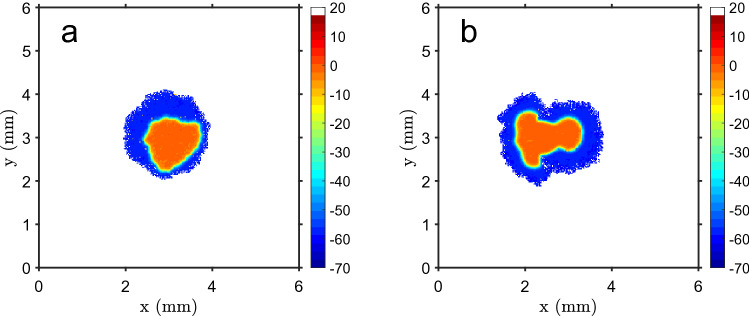
Effect of a carcinogenic event. (**a**) Example of the tissue final state, after 500 cell cycles, ensuing from random depolarization of 40% of the cells at $t=300$ cell cycles, simulating a dispersed carcinogenic event, which increases the tissue size and the depolarized core. (**b**) Final state after a localized depolarized event, a circular depolarized spot, with radius $R=20$ cells, localized to the left of the central tissue region, at $t=300$ cell cycles. This depolarized spot drives tissue growth and gets merged with the initial depolarized core. Color bar: cell membrane potential, in mV (white shows empty space).

**Figure 6:**
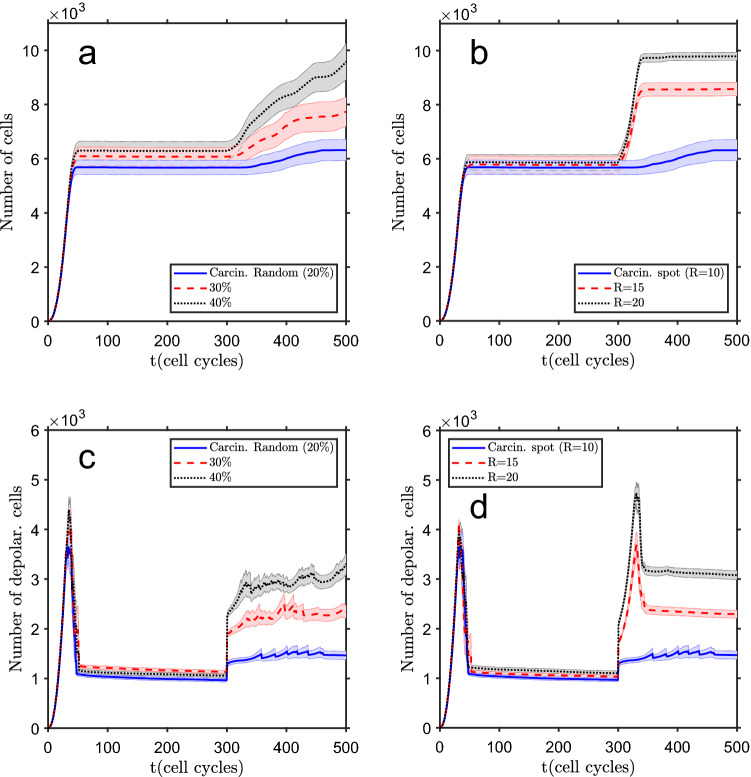
Tissue growth due to a carcinogenic event occurring at $t=300$ cell cycles. Total number of tissue cells as a function of cell cycles (**a**) and number of depolarized cells (**c**) for a dispersed event (random depolarization of a percentage of cells). Total number of tissue cells as a function of the number of cell cycles (**b**) and number of depolarized cells (**d**) for a localized event (depolarization of a spot with radius *R*, in number of cells). The bands show the standard deviation of the mean of $n=25$ simulation runs.

The carcinogenesis process can be simulated, in the present model, by random depolarization of a certain percentage of tissue cells (a dispersed event, like a chemical substance distributed in the tissue) or by localized depolarization of cells (a spot event, like the effect of an ionizing radiation beam). In Fig. 5 an example of both types of events is presented. In the first one an increase of the tissue size, with a considerable larger depolarized core (as compared with the standard conditions shown in Fig. 1a) can be seen. The expanded depolarized core makes the tissue more sensitive to further carcinogenic events, thus having a cumulative effect. In the other situation, there is a second depolarized core, centered at the depolarization spot, connected to the original one. The results obtained for the evolution of the total number of cells and of depolarized cells are shown in Fig. 6, and if the radius of the spot or the percentage of depolarized cells are above a certain limit ($R\sim 10$ cells or a percentage $\sim 20$ %), there is a significant increase of the tissue area, followed by new repolarization, and this process can be repeated in several stages. For a random polarization event, in the left column, the number of cells increases in a smooth way, and, for a higher percentage of depolarized cells, this can be a multi-step process, with the successive emergence of new depolarization spots, seeding new growth surges. For localized carcinogenesis (right column), the system shows a fast growth and then stabilizes. In this case, the number of depolarized cells also shows a quick increase but, due to their polarization induced by communication with neighbor cells, decreases to an approximately constant number.

From the results presented in this section it is clear that it is possible to increase the tissue size and change its shape by cells’ depolarization. This is expected as depolarization allows cells to regain their proliferation capacity and, if they have space available (on the tissue periphery), they can occupy new areas. The two types of carcinogenic events studied, dispersed and localized, lead to different patterns of time evolution, but both increase the depolarized core size. This is a potentially dangerous development because it decreases the threshold for a new carcinogenic event. The accumulation of depolarization events with time can explain the increased probability of developing cancer when getting older^82,83^. In general, the system is resilient to these events, with the result of cells connectivity via gap junctions driving the return to the initial bioelectric conditions when the carcinogenic event is below a certain level (percentage of depolarized cells or depolarized cells spot radius). The cells’ bioelectric properties can be different at the genomic, and then hereditary, level, which would make some persons more susceptible to carcinogenic events and then to develop cancer. In this model, we are not considering the deleterious effects of excessive proliferation on the cellular environment, like hypoxia, decreased pH or increased pressure, which would increase the local cells death rate and open space for further proliferation.

### Cancer can be reversible by cell repolarization

**Figure 7:**
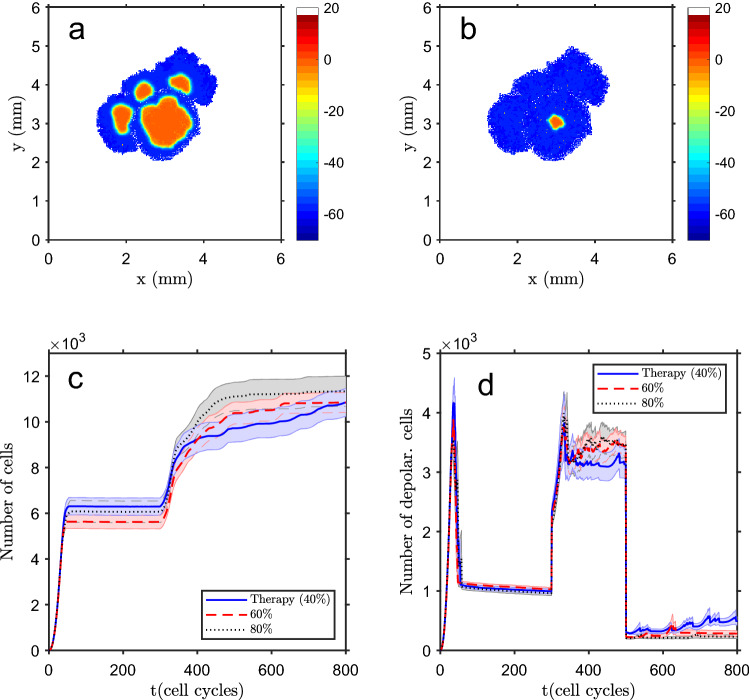
Tumor therapy. After the normal tissue growth, it suffers a dispersed carcinogenic event at $t=300$ cell cycles (random depolarization of 40% of the cells). The tumor grows and a diffuse therapeutic intervention is applied at $t=500$ cell cycles. (**a**) Tissue state at $t=500$ cells cycles, just before the therapy. (**b**) Tissue state at $t=800$ cell cycles (after the random depolarization of 60% of the cells), showing that almost all the tissue is polarized, therefore in a non-proliferative state. Color bar in (**a**,**b**): cell membrane potential, in mV (white shows empty space). Evolution in time of the total number of tissue cells (**c**) and depolarized cells (**d**) for three therapy levels (percentage of randomly repolarized cells). The bands in (**c**,**d**) show the standard deviation of the mean of $n=25$ simulation runs.

One positive side of the depolarization event carcinogenic hypothesis is that it is reversible^64,65,84^, in the sense that the depolarized/proliferative cells can return to the polarized state, which stops tumor growth. This test is shown in Fig. 7. After a carcinogenic event at 300 cell cycles (random depolarization of 40% of the cells), the tumor starts to grow (in this example, as a multi-spot event, shown in Fig. 7a). At $t=500$ cell cycles a therapy is applied, which can be simulated by a chemical substance that diffuses on the tissue, affecting some specific ion channels, and driving random repolarization of 60% of the cells. After this intervention, and due to cells’ connectivity via gap junctions, the tissue becomes almost fully polarized, hence non-proliferative, except for a small depolarized core (Fig. 7b). The evolution in time of the total number of cells and of depolarized cells is shown in Fig. 7c,d, respectively, for different therapy intensities.

As expected, a more intensive treatment (a larger percentage of repolarized cells) leads to a more stable total number of cells and to a lower and more constant number of depolarized cells.

The therapy should be applied locally, as a systemic delivery of ionic channels manipulation drugs can be dangerous for other fundamental processes that rest on membrane potential changes, such as in the nervous system or the heart. The drug can be encapsulated into a vehicle that reacts to particular values of cell membrane potential and delivers its cargo when the correct conditions are met. For instance, voltage-reporter dyes, used for membrane electric potential visualization, already profit from a similar property. On the prevention side, events and molecules that interfere with ionic channels function and/or cell membrane polarization should be studied for potential carcinogenic effects and, eventually, limits on their use should be considered.

## Discussion and conclusions

We developed, adjusted and run a very simple computational model of growth and persistence of an elementary two-dimensional organism, based in some tenets of a Theory of Organisms. Most of the hugely complex mechanisms involved in biological embryogenesis were not considered, with effort concentrated in cells and tissues bioelectric properties. However, even a manifestly oversimplified model can be useful to test some assumptions and to point out new directions of research or different ways to look into complex phenomena. Albeit bioelectric properties are central to this work, it can be adapted to a more realistic biological description, with the addition of other components, as biochemical reactions or mechanical interactions. Organ patterning can be introduced in a simple way by the use of diffusive substances, like the Turing model for biological patterns^85^ or by bioelectric signaling^86,87^. The constraints included are limited and therefore the system doesn’t grow always to the exact same final state, due to the cells’ properties variability. The major constraints are spatial, with competition for proliferation and migration spots, describing the effect of contact inhibition, and a limitation on the number of cell cycles due to successive membrane polarization.

Some of the results obtained with the computational model (a cellular automaton) beget for more experimental results, like determination of cells and tissues bioelectric state (in particular the transmembrane potential) during organs’ or organisms’ development, or their state and changes in mature tissues. Also, polarization and depolarization tests, in particular in the more complex in vivo environment, would provide useful informations about the contribution of bioelectric potentials and currents in non-excitable cells to tissue conditions and activity.

There are some fundamental processes missing in the present model, from cell phenotype changes (differentiation) to all the cell internal biochemical signaling, complex feedback mechanisms, mechanical interactions and many others. Focusing in a very specific feature, the cell and tissue bioelectric properties, and even these in a simplified way, however, allows to study the consequences of some simple hypothesis, as the way cells can keep their proliferative potential, limited by environmental conditions (like space availability) and ion exchange with neighboring cells (modifying the value of membrane potential). It can also be used to investigate the effect of cells’ properties variability on system progression, with stochastic phenomena in cells interaction being responsible for the system evolution and resilience. Finally, it is useful to examine the effect of a perturbation in tissue bioelectric equilibrium, when above some level, on homeostasis and proliferation control, which can be related with carcinogenesis.

The main conclusions from this work on a computational model of organism development and carcinogenesis, based on bioelectric properties, is that the size and shape of an organism can, potentially, be controlled by this mechanism, in particular the progress of membrane electric potential, from depolarization to polarization, and the consequent effect on proliferation capability, and competition for space^88^. One important outcome is that cell proliferation capability is, in principle, reversible, by changes on bioelectric environment, being it the state of neighboring cells or the consequence of an external event. This is important both in health, for organism or tissue development and homeostasis, and in disease, as excessive cell proliferation seen in tumor development. A more detailed knowledge about bioelectric properties of non-excitable cells, and how they can be controlled, can point to new research directions in cancer prevention and therapy, as the tissue state can, in principle, be normalized by regulation of ion channels and pumps, or control of gap junctions’ activity.

The reported model predicts that cells at the central part of a tissue, during its development, are more depolarized, thus proliferative, than the ones at border, as they have gone through a lower number of proliferation cycles. It also predicts that depolarization of healthy cells drives them closer to a tumor like behavior, with increased proliferation, migration, resistance, etc. Further, tumor cells after membrane repolarization intervention should show more normal or, at least, less stable tumoral properties. Another important conjecture is that individual cell mutations are not at cancer origin but these are mainly a consequence of fast and uncontrolled proliferation and of tissue homeostasis deregulation. Finally, perturbation of a tissue bioelectric state, by addition of, for instance, the appropriate channelopathy therapeutic drugs, can lead to development defects. More experimental tests along these lines allow to test the model hypotheses and also to refine its predictive power.

There is a clear need of further experimental efforts to investigate the relation between cells and tissues bioelectric state and properties, such as organism development, organization and carcinogenesis. One area that requires more attention is the study of the influence of carcinogenic events, as ionizing radiation or chemical substances, in the modification of the tissue bioelectric state. Specifically, emphasis should be placed on studying how cell ion channels and pumps, gap junctions and electrical conductance and capacity, are affected by these events. The study of small molecules capable of changing bioelectric properties of cells, to be used in cancer prevention, diagnostic and therapy should be actively pursued as well^89^. Finally, it is now obvious that a theory of organisms’ organization and regulation is fundamental for an informed investigation of organism development and homeostasis, and also of the carcinogenic process and control^11,90^. Finally, further details should be added to the present model, including relevant biochemical and physical processes, for a realistic explanation of tissues organization and development besides the presented oversimplified description.

A simple and focused model to describe a biological system dynamic as complex as organism development and carcinogenesis can, anyhow, suggest new controlled experiments, whose results, interpreted along this new perspective, provide alternative explanations and point novel research directions.

## Data Availability

The matlab code used in the simulations is available from the author upon request.
